# Cortical Activation Patterns Evoked by Temporally Asymmetric Sounds and Their Modulation by Learning

**DOI:** 10.1523/ENEURO.0241-16.2017

**Published:** 2017-04-21

**Authors:** Junsei Horikawa, Hisayuki Ojima

**Affiliations:** 1 Department of Computer Science and Engineering Graduate School of Engineering, Toyohashi University of Technology, Hibarigaoka 1-1, Tempaku, Toyohashi, Aichi 441-8580, Japan; 2 Cognitive Neurobiology, Graduate School of Medical and Dental Sciences, Tokyo Medical and Dental University, 1-5-45 Yushima, Bunkyo-ku, Tokyo 113-8549, Japan

**Keywords:** belt field, primary auditory field, sound discrimination, spatiotemporal activation, time-reversed sound, voltage-sensitive dye imaging

## Abstract

When complex sounds are reversed in time, the original and reversed versions are perceived differently in spectral and temporal dimensions despite their identical duration and long-term spectrum-power profiles. Spatiotemporal activation patterns evoked by temporally asymmetric sound pairs demonstrate how the temporal envelope determines the readout of the spectrum. We examined the patterns of activation evoked by a temporally asymmetric sound pair in the primary auditory field (AI) of anesthetized guinea pigs and determined how discrimination training modified these patterns. Optical imaging using a voltage-sensitive dye revealed that a forward ramped-down natural sound (F) consistently evoked much stronger responses than its time-reversed, ramped-up counterpart (revF). The spatiotemporal maximum peak (*maxP*) of F-evoked activation was always greater than that of revF-evoked activation, and these *maxP*s were significantly separated within the AI. Although discrimination training did not affect the absolute magnitude of these *maxP*s, the revF-to-F ratio of the activation peaks calculated at the location where hemispheres were maximally activated (i.e., F-evoked *maxP*) was significantly smaller in the trained group. The F-evoked activation propagated across the AI along the temporal axis to the ventroanterior belt field (VA), with the local activation peak within the VA being significantly larger in the trained than in the naïve group. These results suggest that the innate network is more responsive to natural sounds of ramped-down envelopes than their time-reversed, unnatural sounds. The VA belt field activation might play an important role in emotional learning of sounds through its connections with amygdala.

## Visual Abstract

**Figure:**
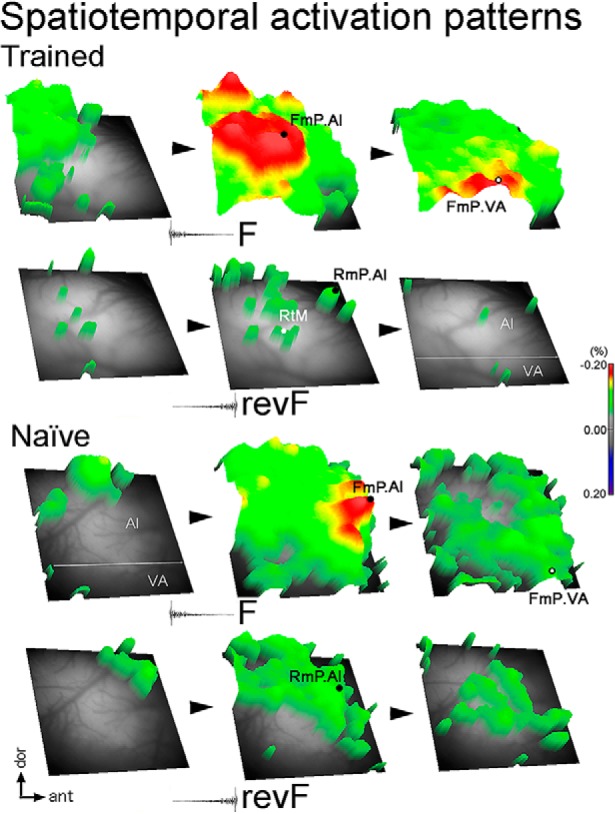

## Significance Statement

Sound is perceived differently when it is played in the forward and reverse directions, despite that the duration and long-term spectrum profiles are identical for these time-reversed sounds. The perceptual differences must derive from the asymmetric processing of spectral transients of sounds and the temporal interactions of neuronal activity elicited by each transient. Spatiotemporal activation patterns can further our understanding of the temporally asymmetric sound processing. We for the first time imaged the distinct cortical activation evoked by a representative pair of temporally asymmetrical sounds and showed differences in the magnitude and location of the activation peaks and their latency from sound onset. Furthermore, sound recognition training enhanced neuronal activity in the belt field presumably involved in perceptual learning.

## Introduction

The temporal variation in the overall sound amplitude, known as the envelope, is one of the critical factors for determining the quality of sounds. Natural sounds are biologically meaningful when they are presented in the forward direction but may be perceived as unfamiliar or peculiar when played in reverse (Licklider and Miller, 1951). Temporally reversed sound pairs are identical in their composition of the long-term spectrum power (Patterson, 1994a,b), but they are perceived differently. For artificial sound pairs, including one with damped envelopes (i.e., starting with a quick attack followed by an exponential decay) and the other with ramped-up envelopes (i.e., starting with a gradual attack followed by a rapid decay; Patterson, 1994a,b), human listeners perceive carrier sinusoids more robustly in the ramped-up version than in the damped version. Similarly, ramped-up sounds are perceived higher in loudness (Irino and Patterson, 1996) and longer in subjective duration (Schlauch et al., 2001). Comparable perceptual differences have been reported for sounds with broadband noises as a carrier (Akeroyd and Patterson, 1995). Previous reports also suggest that there is a minimal duration necessary for the temporal reversal of portions of a spoken sentence to deteriorate its intelligibility (Saberi and Perrott, 1999).

Behaviorally, previous studies have shown the asymmetric perception of ramped-down and ramped-up sounds in animals (Fay et al., 1996; Le Prell and Moody, 2000; Ghazanfar et al., 2001; Ojima and Horikawa, 2016). Rhesus monkeys showed right-ear orienting behavior to conspecific calls, whereas they exhibited left-ear orienting behavior to the time-reversed versions (Ghazanfar et al., 2001). This finding suggests a hemispheric lateralization of the processing of asymmetric sounds. In accordance with behavioral asymmetry, neuronal response preferences for species-specific calls over their time-reversed versions have been revealed in several animal species, including bats (Esser et al., 1997; Medvedev and Kanwal, 2008), songbirds (Margoliash, 1983; Doupe and Konishi, 1991), cats (Gehr, et al., 2000), and monkeys. A larger number of neurons in the marmoset primary auditory field (AI) preferred natural twitter calls than their time-reversed versions (Wang et al., 1995; Wang and Kadia, 2001). In rhesus monkeys, modest asymmetric response preferences were also found for several major vocalizations (Recanzone, 2008).

Except for vocalizations and calls, natural sounds are generally broadband noises and nonharmonic in spectral structure, and their instantaneous spectrum content varies rapidly. These sounds have been largely excluded from research interests, despite their prevalence in our environment. We can easily think of examples, such as knocking on a door, clapping hands, dropping hard objects, and stepping on the floor. The amplitude of these environmental sounds tends to be damped after the initial attack. Thus, the acoustic environment in nature may be biased toward the generation of damped type sounds rather than ramped-up type sounds. These environmental sounds are meaningful when they are played in the normal direction but less so if they are played in the reverse direction. We hypothesized that there should be corresponding biases in the neuronal mechanisms and connectivity for processing such time-reversed sound pairs. To test this hypothesis, we needed to record neural activity globally within the auditory cortex. Therefore, optical imaging was used to examine differences in responses to forward and reversed sound pairs. Considering the identical long-term spectrum content of the temporally asymmetric sounds pairs, we assume that potential asymmetric organization revealed will provide a clue to understanding how the temporal structure is influences the representation of the instantaneous spectrum of complex sounds and evolves over time as determined by the sound spectrogram.

The spatiotemporal pattern of neuronal activities is a fundamental readout of signals processed by sensory cortices (Cohen et al., 1978; Chemla and Chavane, 2010). A voltage-sensitive dye imaging technique can determine a spatiotemporal sequence of activation at a high temporal resolution. Using this technique and a temporally asymmetric sound pair, we aimed at answering the following questions. How different are the spatiotemporal activation patterns evoked by sound pairs with temporally asymmetric envelopes? Can discrimination learning modulate this putative differential processing?

## Materials and Methods

### Behavioral experiment

#### Animals

This study was approved by the local animal care committees at the universities for which both authors worked (nos. 0150209A and 26.7). It is also in accordance with the National Institutes of Health Guide for the Care and Use of Laboratory Animals (NIH publications no. 80-23, revised in 1996) as well as the policies of the Society for Neuroscience. Guinea pigs (Hartley, male; body weight, 350–400 g) were purchased from a commercial supplier (Japan SLC). The animals were first transported to the university of one of the authors for training and then transferred to the university of the other author for imaging experiments. Naïve animals used as the control group were directly transported to the latter institute.

#### Training, training facilities, and sound delivery system

Guinea pigs were trained in a newly developed procedure in which competition was introduced to raise the motivation of animals (Ojima et al., 2012). During the training period, diet was strictly controlled for several days to maintain their body weight at ∼90% of the weight on the day of transportation. The body weight was then gradually increased. The training consisted of two stages. The early-training stage lasted for one week during which one pair of animals was housed in a single home cage within the laboratory. During this period, the animals were fed a small amount of pellets each time a conditioning sound was played through a dynamic speaker (NS-10MM, Yamaha). Feeding was arbitrarily timed within the sound-on period. After this acclimation period, the animals underwent daily training in which they were automatically fed at a constant delay of 1.6 or 3.2 s after the offset of a conditioning sound. The late-training stage lasted for another one week. During the first 3-4 d of this stage, the cage-mate pair was placed in a training arena (50 × 50 cm) within a sound-attenuating chamber and then trained with the training sound set (see below for details). During the remaining 3 d, the paired animals were separately trained in an otherwise similar manner to the preceding days. On the next day following the late training, each animal was individually subjected to a one-chance trial of generalization testing (see below for details). Naïve animals (control group) were not trained, being housed for one or two weeks in a cage in an acoustic environment similar to the one used for the behavioral training. The husbandry staff was careful to not produce sounds similar to the conditioning sound while feeding or caring for the naïve animals.

The sound-attenuated chamber contained a training arena made of metal mesh (acoustically transparent), one monitor microphone (F-320, Sony), three video cameras (WAT-204CX, Watec and SH-6C, WTW), one custom-made infrared motion detector, and two loudspeakers (NS-10MM, Yamaha) that were positioned 1.7 m above the arena and 1 m from each other. The loudspeakers were used for sound reproduction. The sound delivery system was calibrated 30 cm above a food saucer using a half-inch condenser microphone (7012, ACO) and compensated at 1/3-octave intervals from 80 Hz to 12.5 kHz (Q2031B, Yamaha). Sounds were reproduced at 68 ± 6-dB SPL after amplification through a power amplifier (N220, Sony).

#### Training sounds and testing procedures

**Figure 1.**
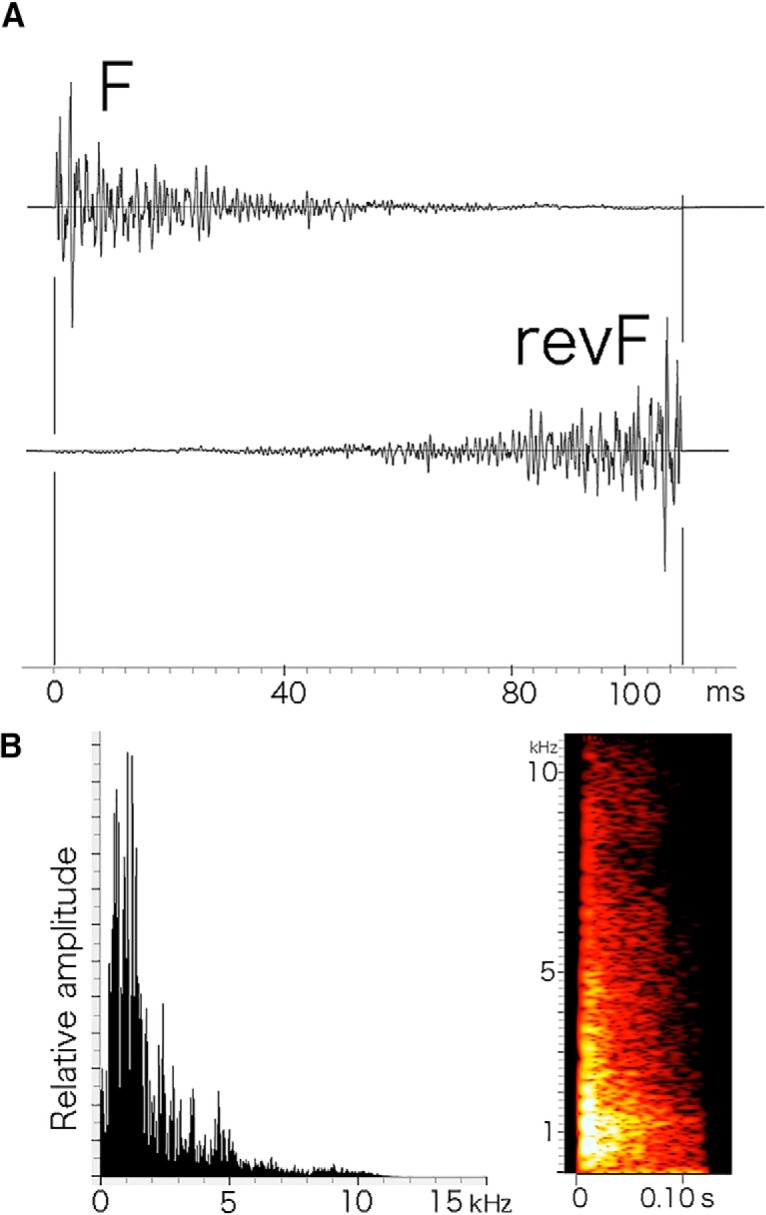
The stimulus sound segments, F and revF, used for behavioral training and optical imaging. Sound stimuli are presented to animals as a train of four-time repeated F or revF segments in the optical imaging. ***A***, The F segment is a normal natural sound (footstep sound) and the revF segment is its time-reversed version. ***B***, The power spectrum (left) and sonogram (right) of the F segment. Note that the F and revF segments have an identical long-term power spectrum according to the Fourier transformation (Patterson, 1994a,b).

The sounds used for training and testing were produced with a specialized software (Amadeus Pro, http://www.hairersoft.com/) by editing natural sounds that were originally generated by stepping on the laboratory floor, clapping hands, hitting a plastic cage, hitting a metal can, scratching a metal mesh, and jingling keys. One representative sound segment from each of these natural sounds was duplicated 9-12 times to make respective multi-segment sound trains. The ramped-down forward natural sound (F) used for conditioning was a footstep sound. Specifically, the conditioning sound was 4.7 s in duration and composed of 10 F segments with an identical spectrum except for the amplitude, which varied within a range of ±6 dB. The intersegment intervals were fixed to a relatively long value of ∼0.5 s. Each F segment (110 ms in duration) had a ramped-down envelope with its peak pressure at 5 ms soon after sound onset and a spectral bandwidth extending up to ∼12 kHz. Much of its energy was distributed in the lower frequency region (Fig. 1). Other sounds were constructed in a similar way and used as distracters (nontarget sounds, NTs). The training sound set, which was played several times per day, consisted of six different sounds, including one F train and five NT trains (intersound intervals of ∼60 s), presented in a randomized order for different trials, sessions, days, and animals. For the behavioral testing and imaging experiments, each segment of the F stimulus train was simply reversed in time to make the time-reversed version of F (revF; Fig. 1).

### Optical imaging experiment

#### Surgery

Imaging was conducted from both hemispheres. After completion of imaging from one hemisphere, the second craniotomy was started for the other hemisphere. Animals were initially anesthetized with a mixture of ketamine and xylazine (i.m., 80 mg/kg, Ketalar, Daiichi-Sankyo, and 25 mg/kg, Selactar, Bayer Yakuhin, respectively), placed under a measuring microscope in a soundproof room, and artificially ventilated after injection of the muscle relaxant pancuronium bromide (i.m., 1 mg/kg, Myoblock, MSD). The auditory cortex exposed by craniotomy was stained with the voltage-sensitive dye RH795 (Invitrogen) for 60–90 min. Heart rate and body temperature were continuously monitored. A supplemental dose of anesthetics (25 mg/kg Ketalar and 10 mg/kg Selactar) and the muscle relaxant (1 mg/kg Myoblock) was administered every 60–120 min. Animals were euthanized with pentobarbital (i.p., 60 mg/kg, Somnopentyl, Abbott) or ketamine (intracardiac, 160 mg/kg) after completion of the experiment.

#### Optical imaging procedure

**Figure 2.**
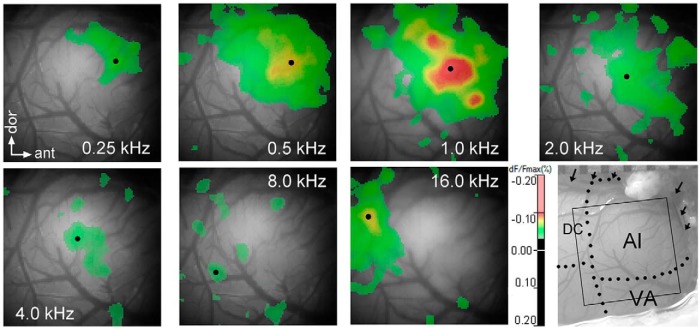
Tonotopic activation of the primary auditory cortex by a set of pure tones. Pure tones with a 200-ms duration and 5-ms onset/offset cosine ramps are reproduced at frequencies of 0.25, 0.5, 1, 2, 4, 8, and 16 kHz at 75-dB SPL in otherwise the similar manner to the asymmetric sound pair. On a conventional light micrograph (lower right), covering the anterior part of the guinea pig’s AI, the optical image frame (square) is superimposed. The approximate borders between the AI and DC field and those between the core AI and the belt VA are depicted by dotted lines. Thick blood vessels (a set of arrows) course along the pseudosylvian sulcus. Dots point to the maxima of activation evoked by different tones. For the tone-evoked activation maps, refer to Figure 3.

The auditory cortex contralateral to sound stimulation was epi-illuminated by a 480- to 580-nm fluorescent light with the ipsilateral ear clogged (Horikawa et al., 1996). Light signals emitted from the cortex (>620 nm) were recorded with a CMOS camera (MiCAM Ultima, www.brainvision.co.jp) attached to the measuring microscope. The camera consisted of a sensor array of 100 × 100 channels, which corresponded to a 5 × 5 mm image frame. The image frame was captured every 2 ms with focusing at 300 μm below the cortical surface. The AI was positioned approximately at the center of the image frame (Fig. 2, lower right) according to the pseudosylvian sulcus.

Sensor-detected light signals may include false-positive noises caused by fine movement derived from pulsation. To eliminate this artifact, the onset of each of the sound-on and silence periods was phase-locked to the simultaneously recorded electrocardiogram (ECG). Then, differential signals, extracted by subtracting the signals recorded during the silence period from those recorded during the sound-on period for each presentation of the sound unit, were averaged across its four presentations to obtain trial-unique differential signals (dF). Signals recorded at the periphery of image frames were tended to be less accurate because of weak light emission from the curved surface of cortex. Therefore, signals obtained from the marginal zone equivalent to a five-channel width from each side of the image frame were excluded from quantitative analyses.

#### Sound stimulation during optical imaging

Sound stimulation was controlled with a digital sound generation system (System 3, www.tdt.com) on a PC platform computer. A stimulus unit (∼4 s in duration), which consisted of (1) the sound train (sound-on period, 2 s) and (2) the equal period of silence immediately after the sound-on period, was digitally generated. One sound train contained four segments of either F or revF (intersegment intervals, 0.5 s). Delays of the onset of the stimulus units were temporally adjusted so that the timing of the amplitude maxima of F and revF segments had the same latency from imaging onset; namely, the first F segment started 110 ms after imaging onset, while the first revF segment started 10 ms after imaging onset. The stimulus unit was consecutively repeated four times for averaging.

Illumination was started 3-4 s earlier than the sound onset, and thus the illumination period per trial was ∼20 s in duration. For a given hemisphere, each of the two stimulus sound types, one containing F segments and the other containing revF segments, was presented once at a silent and dark interval of ∼100 s. This relatively short intertrial interval together with the relatively short illumination period minimized the signal attenuation caused by photobleaching. The presentation order of F and revF stimulus types were randomized from hemisphere to hemisphere and this also eliminated the possibility of time-lag-based differences in signal attenuation between the different stimulus types. Sounds were reproduced at an average of 75-dB SPL through a loudspeaker (MSP-5A, Yamaha) placed 10 cm from the orifice of the animal’s external auditory meatus. The spectrum-power specification of the system was compensated to match between behavioral training and optical imaging.

#### Signal processing

**Figure 3.**
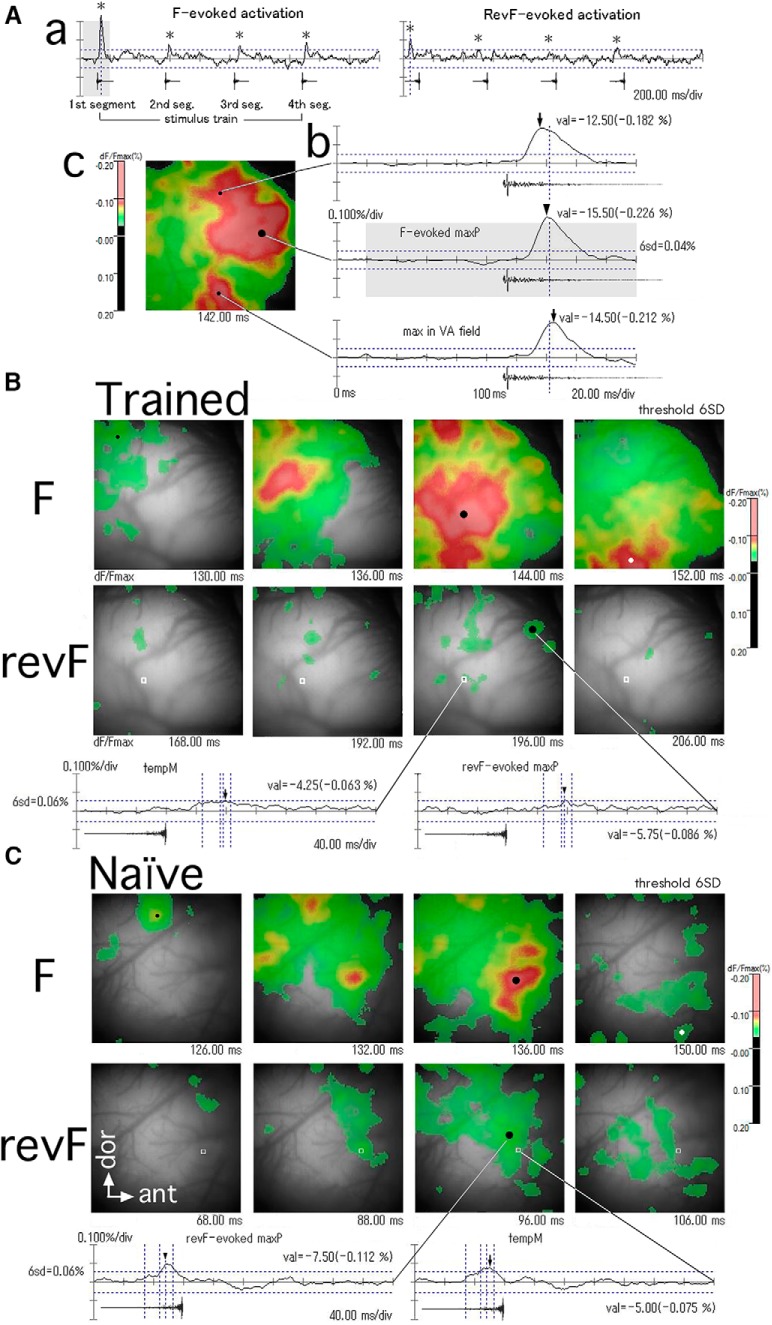
Temporal and spatial patterns of activation evoked by the temporally asymmetric sound pair. ***A***, Differential optical response signals (dF/Fmax, %) recorded at single channels and an activation map generated from the signals recorded across all channels. ***a***, Traces of the response signals averaged across four-time repeats of the stimulus sound train which consists of four identical segments of either F (left) or revF (right), as shown below each response trace. Note that the F- and revF-evoked response traces are different even if they are recorded from the same channel. The temporal trace of responses typically shows 4 transient positive deflections that are time-locked to the individual sound segments (asterisks). Note that depolarization takes negative dF/Fmax values that are represented as the upward deflection in the response traces. The shaded portion of the response trace in ***a*** is enlarged in the middle trace in ***b*. *b*,** Traces of the F-evoked response signals recorded at three different locations show the *tempM*s (arrows and arrowhead) in amplitude at different delay times after the sound onset. ***c***, A 2-ms image frame, recorded at the time of the dotted vertical line in ***b***, shows the map of activation that is above the threshold (i.e., 6 SD of the mean of spontaneous activities). The suprathreshold signals are color-coded according to their magnitude (scale bar). Each image frame has a spatial peak, and the largest of these peaks across all the frames recorded for a given trial is designated as the trial-unique *maxP*. The *maxP* of activation within the AI is indicated by the large dot in the map and corresponds to the peak (arrowhead) of the trace shown in ***b***. The time when the image frame is recorded (imaging onset is 0) is shown just below the activation map. Image frames have the dimensions of 5 × 5 mm. ***B***, ***C***, Temporal sequence of activation maps evoked by the first F and the first revF segments (upper and lower panels, respectively) during the period of activation in the trained (***B***) and naïve (***C***) animals. The 6 SD of the mean of spontaneous activity values is used as the threshold. Large black dots indicate the *maxP*s within the AI. Small black dots in the F panels show the initial activation peak during the activation period. White dots in the F panels point to the *maxP* of F-evoked activation within the VA. Two temporal traces of the revF-evoked activation, one recorded at the channel of the revF-evoked *maxP* (large black dots in the revF panel) and the other recorded at the channel corresponding to the F-evoked *maxP* (open white squares), are shown below traces. Arrowheads and arrows indicate the *tempM* at the respective recording channels (note that the arrowhead on the trace recorded at the large black dots corresponds to the revF-evoked *maxP*). Dotted vertical lines on the revF-evoked traces indicate the time when different image frames are recorded.

**Figure 4.**
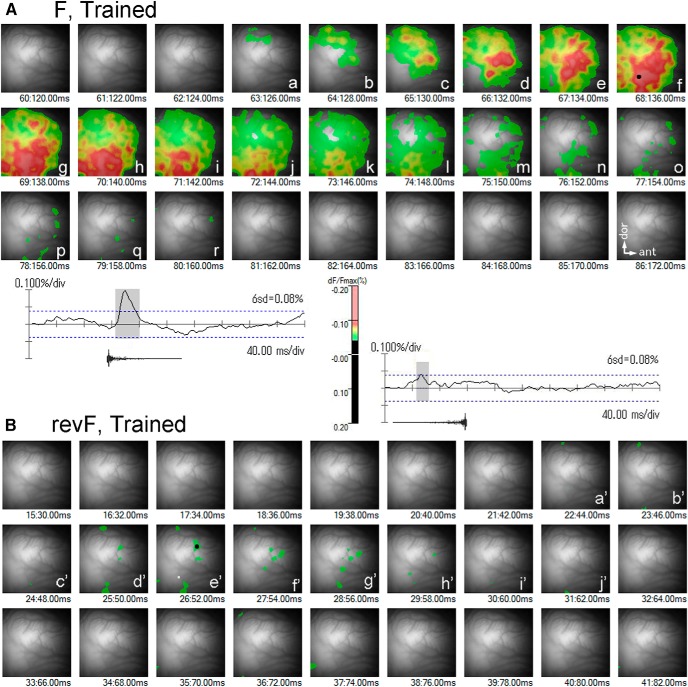
Full-time course of trial-unique activation maps in the trained animal. The activation maps evoked by the 1st segments of F (***A***) and its time-reversed revF (***B***) in a trained animal are chronologically arranged at 2-ms intervals. The temporal traces of response signals recorded at the F- and revF-evoked *maxP*s (large dots in ***Af*** and ***Be'***) are shown below or above the respective frame sequences. In the revF-evoked activation map, the location where the F-evoked *maxP* is evoked is indicated by the open white square (***Be'***). The activation maps labeled with lower-case letters are derived from the hatched portions of the response traces. All image frames have the dimensions of 5 × 5 mm.

**Figure 5.**
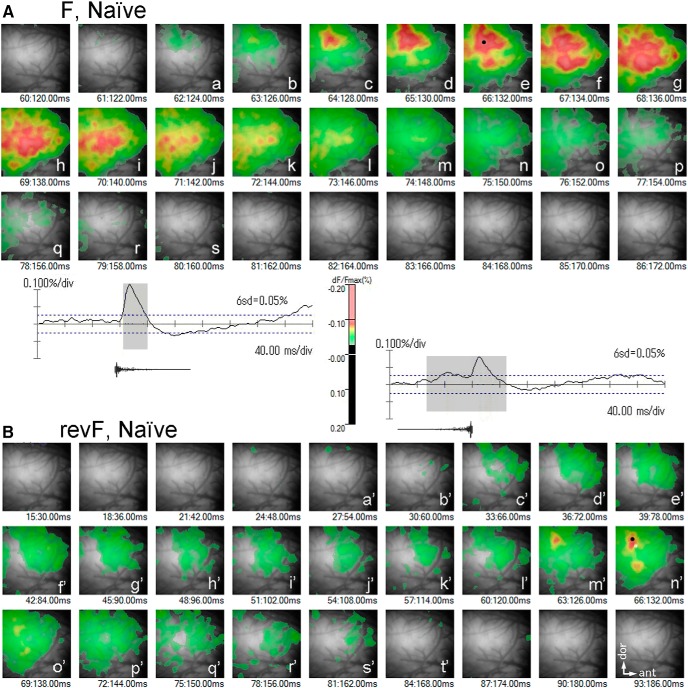
Full-time course of trial-unique activation maps in the naïve animal. The activation maps evoked by the 1st segments of F (***A***) and its time-reversed revF (***B***) in a naïve animal are chronologically arranged at 2- and 6-ms intervals, respectively. The temporal traces of response signals recorded at the F- and revF-evoked *maxP*s (large dots in ***Ae*** and ***Bn'***) are shown below or above the respective frame sequences. In the revF-evoked activation map, the location where the F-evoked *maxP* is evoked is indicated by the open white square (***Bn'***). The activation maps labeled with lower-case letters are derived from the hatched portions of the response traces. All image frames have the dimensions of 5 × 5 mm.

For each channel (x,y), the dF of the background image captured just before the imaging onset was normalized to the maximum light intensity (Fmax) of the background image in each trial. If this normalized base intensity at a given channel, F(x,y), was larger than 0.25, the differential signal calculated for this channel, dF(x,y), was modified to a value of dF(x,y)/F(x,y) (designated as dF/Fmax, %) as an optical response signal. If the F(x,y) was equal to or smaller than 0.25, the dF(x,y) was assigned a value of zero. The dF/Fmax was measured at all the channels of each of the consecutive image frames for a given trial. Thus, for any channel, the temporal change of the dF/Fmax signal was defined as a continuous trace with its amplitude varying as a function of time (Fig. 3*A*a,*b*). This signal trace for a given channel (or location) was peaked at a certain time after sound onset, and this peak was designated as the temporal maximum (*tempM* or *tM*). If there was more than one peak at different times, the earliest one was adopted for a given trial. The dF/Fmax measured for all the channels of each of the consecutive image frames was also used to construct a trial-unique sequence of activation maps (Figs. 4, 5). Note that depolarization takes negative dF/Fmax values and are represented as upward deflection in the response traces.

#### Cortical activation maps

On each image frame, cortical activation was mapped (Fig. 3*A*c,*B*,*C*) on the basis of the magnitude of normalized differential signals (dF/Fmax) across all the channels. For mapping, only depolarization was depicted. First, the signals were spatially filtered with an algorithm of averaging 7 × 7 neighbors. The temporal trace of the signal at each channel was then temporally filtered at 4 and 40 Hz for high pass and low pass, respectively. The largest signal value within each image frame was chosen as a spatial peak, and the largest of these spatial peaks across all image frames per trial was designated as the maximum peak (*maxP or mP*; Fig. 3, large dots). If there was more than one channel showing the same *maxP* value, signal values of the channels surrounding each peak channel were separately averaged and the one with the largest mean was chosen as the peak. Thus, the *maxP* represents the trial-unique spatiotemporal maximum evoked by a particular stimulus type, and can be defined separately for each of distinct cortical fields.

A portion of the signal trace recorded for 100 ms before the onset of the first F segment reflects the period of spontaneous activity. Signal values of every 2-ms bin within this period of trace recorded at the *maxP* location were averaged to obtain the spontaneous activation level for a given hemisphere. The 6 SD of this mean was used as the threshold to illustrate both the F- and revF-evoked activation maps, in which the suprathreshold channels were color-coded depending on their signal magnitudes. They were then superimposed on a black-and-white cortical surface image as shown in Figure 3. A set of pure tones was applied to reconstruct a coarse tonotopic map (Fig. 2), usually immediately after capturing the response signals to the asymmetric sound pair.

### Data processing

#### Behavioral data analysis

Behavioral performance was assessed by the initiation of behavioral reactions (BhRs), i.e., distinctive conditioned motion characterized by a quick head swaying combined with neck extension at the food saucer and/or circling the food saucer. The BhRs were easily discriminable from spontaneous motion at the food saucer or movement toward the saucer based on the restlessness and quickness of the animal. Trials were defined as positive only if the two following criteria were fulfilled: (1) animals initiated the BhR during the sound train-on period, and (2) they continued the BhR throughout a period of time up to the feeding moment for the F trials or the corresponding time for the revF trials.

#### Imaging data analysis

Stimulation with the sound containing four segments as a stimulus train evoked time-locked deflections on a dF/Fmax signal trace (Fig. 3*A*a, asterisks). Response signals evoked by only the first segment of the stimulus trains were used for the present analyses. This eliminated potential stimulus adaptation effects and alleviated the gradual deterioration in the ECG-based artifact cancellation power. Calculations were made for the following purposes and subjected to statistical analyses (see statistical Table). (1) To evaluate effects of the temporal asymmetry of sound envelopes on the response strength, the trial-unique *maxP*s of activation were compared between the paired sounds. They were also compared between the animal groups to assess learning effects. (2) To evaluate whether a neuronal population maximally activated by the forward sound was also activated maximally by its time-reversed version, the spatial separation between the *maxP*s evoked by the paired sounds was compared with the spontaneous separation between the 2 *maxP*s evoked by repeating the forward sound twice. (3) To evaluate effects of the temporal asymmetry of sound envelopes on the activity of a neuronal population, the *tempM* of activation evoked by the reverse sound (RtM) at the location where a hemisphere was maximally activated (i.e., F-evoked *maxP*) was normalized to this hemisphere-unique *maxP*. These normalized values (RtM at FmP) were compared between animal groups. (4) For the temporal shift of activation, the activation period during which the suprathreshold activation was evoked was sequentially divided into four phases, and the spatial activation maps representing each of these four phases were chosen to illustrate the temporal activation pattern. Since the ventroanterior belt field (VA) was activated during the last phase of the response period, the *maxP* of the VA was defined. For comparisons of the normalized VA activation, the ratios of the *maxP* within the VA relative to the *maxP* within the AI were calculated for individual hemispheres and compared between animal groups.

#### Statistics

The McNemar’s test was used for nonparametric statistical comparisons between the number of animals exhibiting BhRs to the F and revF sound pair. For each hemisphere, a paired *t* test was used to compare the magnitudes of optical response signals evoked by F and revF. An *F* test was used to evaluate the unevenness of the delay time from the sound onset to the *maxP* between animal groups. The Welch’s test was used to compare signal amplitudes between animal groups. To statistically determine if the position of F-evoked activation peaks was spatially distinct from the position of revF-evoked activation peaks, we used the Welch’s test to compare the distance between these two peaks to the distance between the activation peaks obtained by repeating F twice. The comparison of the normalized VA activation between different animal groups was tested with the Welch’s test. The number of hemispheres that showed peaked activation within the VA was compared between the animal groups using the Fisher’s exact probability test.

## Results

In this section, results of behavioral observations are described first. Following it, peak values of the response signals evoked by the F and its time-reversed counterpart (revF) under anesthesia are presented. Then, spatial activation patterns based on the separation of the trial-unique *maxP*s evoked by the F and revF within the AI are shown. For learning effects, the magnitudes of F- and revF-evoked *maxP*s and their relative magnitudes (contrasts) are compared between the trained and naïve animal groups. Finally, for corticocortical propagation of peaked activation to the belt field, the relative magnitudes of the F-evoked *maxP* within the VA to that within the AI are compared between the groups.

### Behavioral tests with the temporally reversed sound pair

In the trained group, animals were conditioned to be rewarded on F sounds only. During the training period, especially in the late-training stage, it was common to observe conflict behaviors such as keeping their body over the food saucer to block the competitor’s approach to it and/or inserting their snout at the orifice of the food hopper to interfere with competitor’s food intake (Ojima and Horikawa, 2016). Such aggressive behavior was never observed for naïve animals. Behavioral tests showed that all of the trained subjects (*n* = 11) responded with BhRs to the F stimulus train, but none of them responded to the revF train except for one subject (McNemar’s test, *p* = 0.002; Table 1). In contrast, naïve subjects (*n* = 11) did not respond to either stimulus sound train of F or revF segments (McNemar’s test, *p* = 1.0; Table 1). These results indicate that the guinea pig can discriminate temporally asymmetric sound types.

**Table 1.** Comparison between behavioral performances evoked by different sound types

Animal group	Number ofanimals used	Response to both F and revF (number of animals)	Response to F but not to revF (number of animals)	Response to revF but not to F (number of animals)	Response to neither F nor revF (number of animals)	Comparison betweensound types	
						Type of test	*p* values
Trained	11	1	10	0	0	McNemar test	0.00195
Naïve	11	0	0	0	11	McNemar test	1.000

### Identification of the AI and belt fields of the guinea pig

Optical response signals were obtained from 12 hemispheres of 8 trained guinea pigs and 12 hemispheres of 6 naïve guinea pigs. These 24 hemispheres were used to compare the *maxP*s of AI activation. For the *maxP*s of VA activation, hemispheres that had bleeding locally from the dura mater near the ventral edge of the image frame were eliminated (18 hemispheres used). On the basis of the mirror-symmetric tonotopic organization of cortical activity to the pure tone set, the border between the AI and dorsocaudal cortex (DC) was estimated. The response latencies and temporal activation patterns were used to estimate the approximate border between the AI and VA (Wallace et al., 1999). In general, the ventral one-fifth zone of the image frame except for a narrow posterior zone (i.e., DC) corresponded to the VA (Fig. 2, lower right). Domains of the pure tone-evoked activation gradually shifted more posterior across the AI as the frequency increased, with the tendency for its anterior part to be activated more widely than its posterior part (Fig. 2). This may be consistent with the dominance of lower frequencies in the frequency spectrum of the guinea pig’s communication calls (Berryman, 1976) and also with the overrepresentation of lower frequencies within the guinea pig’s AI (Wallace and Palmer, 2009).

### Cortical activation evoked by F and revF segments in the core field

#### Optical response signals evoked by F

**Figure 6.**
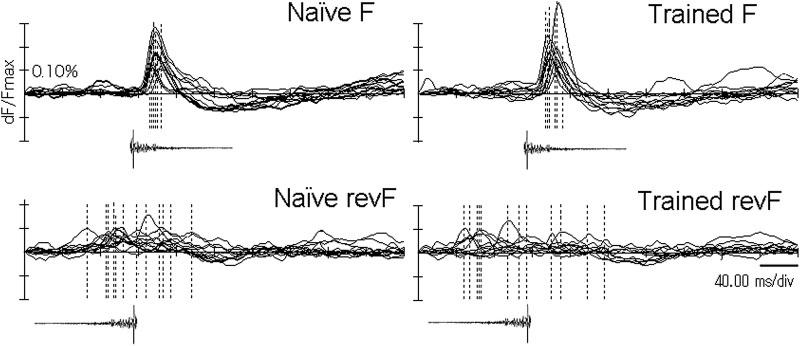
Trial-unique response traces recorded at the *maxP*. Temporal traces of the response signals (dF/Fmax, %) evoked by the first F segment at the channels where the spatiotemporal *maxP* within the AI is evoked. The sound waveforms below traces show the delay times and duration of the stimulus sounds (F and revF). The time of *maxP* is indicated by the dotted line for each trace. F, normal natural sound. revF, time-reversed version of F.

**Figure 7.**
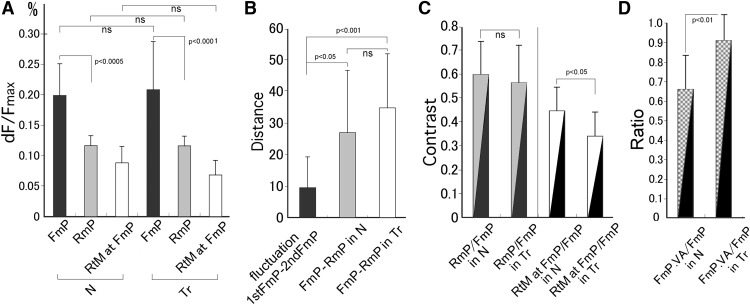
Quantitative comparisons of the peaks of sound-evoked signals between the F and revF stimulation and between the animal groups. ***A***, The trial-unique *maxP*s of activation within the AI (*mP*) are compared between the F and revF stimulation (FmP and RmP) and between the naïve and trained (N and Tr) animal groups. The channel-unique *tempM*s (*tM*) of activation evoked by the revF at the location where the F-evoked *maxP* within the AI is recorded (RtM at FmP) are compared between the animal groups. ***B***, Coordinate-based Euclidian distances between the F- and the revF-evoked *maxP* within the AI (FmP-Rm*P*) are compared between the trained (Tr) and naïve (N) animal groups. These distances for the different animal groups are also separately compared with the spontaneous separation distance between the 2 *mP*s obtained by repeating the F stimulation twice (1stFmP-2ndFmP; i.e., the internal fluctuation). ***C***, Ratios (or Contrasts) of the revF-evoked *maxP* relative to the F-evoked *maxP* within the AI (RmP/FmP) are compared between the different animal groups (left). Similarly, the ratios of the revF-evoked *tempM* at the location of the F-evoked *maxP* relative to the F-evoked *maxP* (RtM at FmP/FmP) are compared between the animal groups (right). ***D***, Ratios of the F-evoked *maxP* within the VA relative to the F-evoked *maxP* within the AI (FmP.VA/FmP) are compared between the naïve (N) and trained (Tr) animal groups. Error bars indicate the standard deviation. ns, not significant.

For the trained animal group, a single F train sequentially evoked four sharp positive deflections on the signal trace at the activated channels (Fig. 3*A*a, left). The channel-unique *tempM* of activation evoked by the first F segment was gradually delayed as recording sites were spatially shifting from the dorsal to ventral region (Fig. 3*A*b). The delay time of the *maxP* of F-evoked activation after the onset of imaging (Fig. 6) was relatively constant across different hemispheres (24–42 ms, 28.3 ± 5.2 ms, SD; Fig. 7; Table 2).

**Table 2.** Comparison between sound onset-to-maximum peak latencies (in ms) for different animal groups

Sound type	Animal group				Comparison between animal groups	
	Trained	Number of hemispheres used	Naïve	Number of hemispheres used	Type of test	*p* values
F	28.3 ± 5.2	12	26.3 ± 4.5	12	Welch test	0.323
revF	86.2 ± 50.2	12	111.5 ± 36.2	12	Welch test	0.171

**Figure 8.**
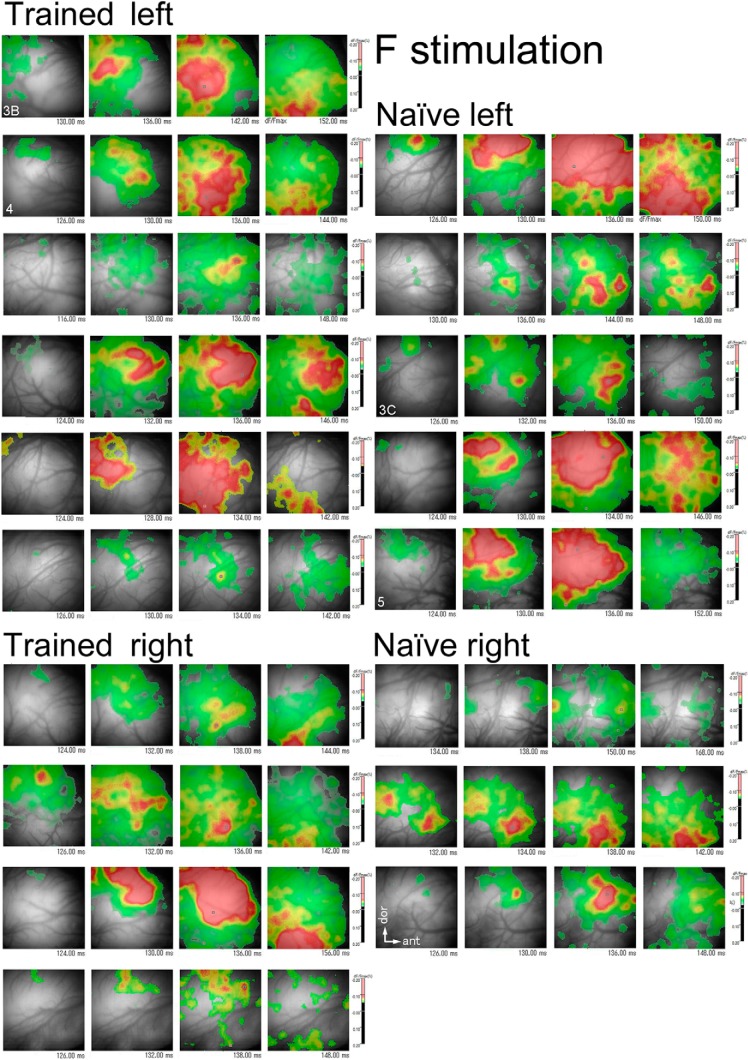
Temporal sequence of the F-evoked activation maps. The activation period during which the F-evoked response signals (dF/Fmax, %) are above the threshold (6 SD of the mean of spontaneous activity values) is divided into four consecutive phases. The activation maps representing each of these phases are chronologically shown (from left to right) in a four-frame panel for a given hemisphere. All hemispheres used for the VA activation analysis are shown. The response signals are color-coded (scale bar, %) according to their magnitude. All scale bars have a magnitude range of -0.20-0.20%, and signal values beyond this range are converted to the range maximum, -0.20%. Domains of the peaked activation (red region) evoked by F stimulation tend to spread into the ventral one-fifth zone of the image frame (corresponding to the VA) and cross its ventral side more frequently in the trained than in the naïve group (Figure 9). Quantitatively, the *maxP*s of VA activation normalized to the *maxP*s of AI activation are significantly larger for the trained than for the naïve group (Fig. 7*D*). Hemispheres used in other figures are labeled with the white numbers in the left frame of panels. All maps are oriented in the same way (white arrow).

A spatial activation map was generated from the signals of all channels of a single image frame at the threshold of 6 SD of the spontaneous activity mean (Fig. 3*A*c). As shown in the Figure 3*B*, upper panel, time course of activation evoked by the first F segment demonstrates that the domains of activation was consistently evoked initially in the dorsal region of the AI (small black dot representing the local peak; Fig. 3*B*, upper panel, left frame; Nishimura et al., 2007). The peaked activation (red/pink domains) shifted ventrally/anteroventrally in a few tens of milliseconds along an axis roughly perpendicular to the anterocaudal frequency gradient as the major domain of activation increased its amplitude and extent across the AI. The trial-unique *maxP* (Fig. 3*B*, upper panel, large black dots in the third frame) was typically found within the strongly activated domain in the AI. Interestingly, the peaked activation extended further ventrally/anteroventrally and shifted to the field ventral to the AI (i.e., VA) in additional several milliseconds with little attenuation of its peak amplitude (Fig. 3*B*, upper panel, small white dot in the right frame). A complete temporal sequence of activation was shown for another hemisphere in Figure 4*A*. The similar patterns of F-evoked activation were observed for almost all hemispheres (Fig. 8, left column).

For the naïve animal group, the delay time of the *maxP* of F-evoked activation (Fig. 6) was also constant across different hemispheres (22–34 ms, 26.3 ± 4.5 ms, SD; Fig. 7; Table 2), and not statistically different from the trained animal group (Welch’s test, *p* = 0.323; Table 2). As in the trained animal group, the peaked activation (red/pink domains) evoked by the first F segment was initiated in the dorsal region of the AI (Fig. 3*C*, upper panel, small black dot in left frame), shifted ventrally/anteroventrally to the central region of the AI where it reached the *maxP* (large black dot), and propagated further ventrally to the VA. However, compared with the trained group, the peaked activation within the VA appeared to be less frequently generated and have smaller peak amplitudes (Fig. 3*C*, upper panel, small white dot in the left frame). A complete sequence of the activation was shown for another hemisphere in Figure 5*A*. Similar patterns of the AI activation were observed for most of the naïve hemispheres (Fig. 8, right column).

#### Optical response signals evoked by revF

**Figure 9.**
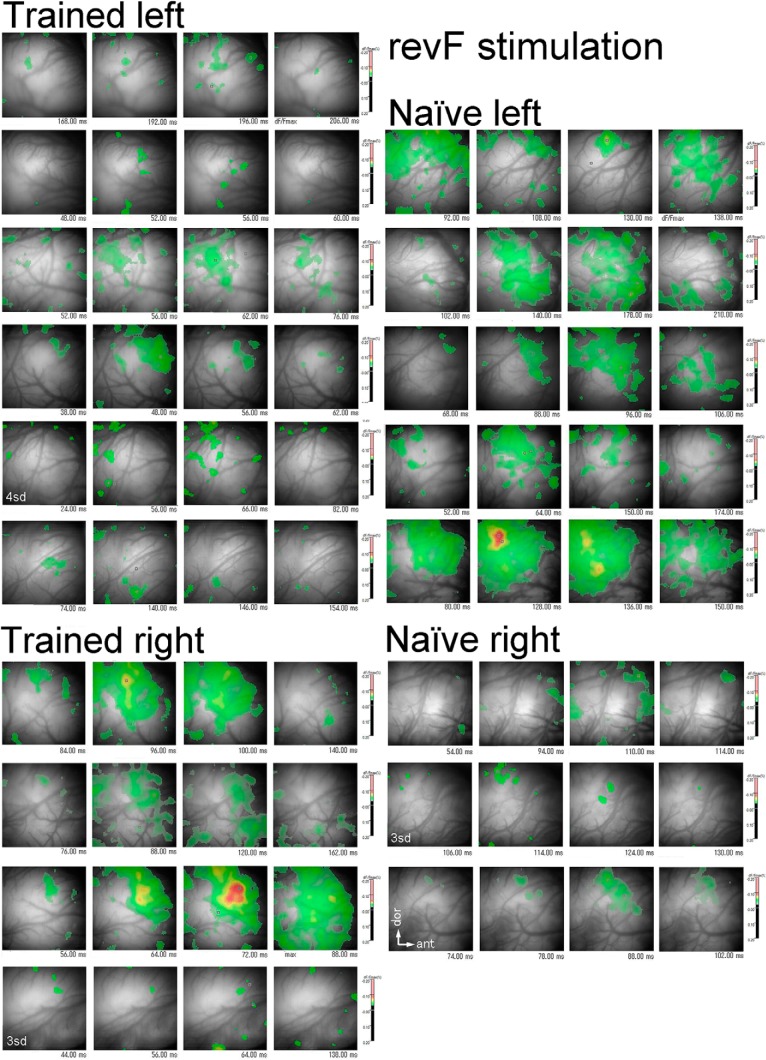
Temporal sequence of the revF-evoked activation maps. The activation period during which the revF-evoked response signals (dF/Fmax, %) are above the threshold (6 SD of the mean of the spontaneous activity values, but the 4 or 3 SD for some hemispheres as labeled directly) is divided into four consecutive phases. The hemisphere-unique activation maps representing each of these phases are chronologically arranged (from left to right) in a four-frame panel for a given hemisphere. All hemispheres used for the VA activation analysis are shown. The response signals are color-coded (scale bar, %) according to their magnitude. All scale bars have a magnitude range of -0.20-0.20%. Activation evoked by the revF stimulation is generally weak except for three hemispheres that have the peaked activation (small red domains) within the AI. However, none of the revF-stimulation evokes the peaked activation within the VA. The panels at the corresponding location of Figures 8 and 9 are of the same hemisphere. All maps are oriented in the same way (see white arrows).

Activation patterns evoked by the revF were markedly different from those evoked by the F in the temporal and spatial dimensions. The revF-evoked activation was generally weak (*n* = 15) or not evident (*n* = 3, namely 2 trained and 1 naïve hemispheres) at the threshold used for the F-evoked activation maps (i.e., 6 SD of the mean of spontaneous activity; Fig. 9). Patterns of the weak activation varied from hemisphere to hemisphere, with some showing dispersed small domains of weak activation (Fig. 3*B*, lower panel) and other showing one major and several minor domains of weak activation (Fig. 3*C*, lower panel). For the hemispheres in which activation was not evident at the 6 SD threshold (see above), lowering the threshold to the 3 or 4 SD exhibited the pattern of dispersed small activation.

The time courses of activation were also markedly different from those of F-evoked activation. In general, the revF-evoked activation domains did not move systematically from the dorsal to the central region of AI (Fig. 3*B*, lower panel) or from the AI to the VA field (Fig. 3*C*, lower panel) during the activation period (Fig. 9). The mean latency from the sound onset to the *maxP* evoked by the first revF segment within the AI (Fig. 6) was significantly longer than that evoked by the F segment both in the trained (38–186 ms, 86.2 ± 50.2 ms, SD; *p* = 6.46E-04; Table 3) and naïve (54–170 ms, 111.5 ± 36.2 ms, SD; *p* = 4.84E-08; Table 3) group, with no significant difference between the groups (*p* = 0.171; Table 2). Variation in the latency was also significantly greater for the revF stimulation than for the F stimulation both in the trained (*p* = 2.31E-0.8; Table 3) and in the naïve groups (*p* = 3.07E-09; Table 3).

**Table 3.** Comparison of sound onset-to-maximum peak latencies (in ms) and their variations between different sound types

Animal group	Number of hemispheres used	Sound type		Comparison of means between sound types		Comparison of variations between sound types	
		F (mean ± SD)	revF (mean ± SD)	Type of test	*p* values	Type of test	*p* values
Trained	12	28.3 ± 5.2	86.2 ± 50.2	*t* test	6.46E-04	F-test	2.31E-08
Naïve	12	26.3 ± 4.5	111.5 ± 36.2	*t* test	4.84E-08	F-test	3.07E-09

### Spatial segregation of peaked activation and comparisons between animal groups

We examined the separation distances between the cortical locations of F- and revF-evoked *maxP* within the AI. The coordinate-based mean Euclidian distance was 26.8 ± 21.3, SD for the naïve and 33.6 ± 15.8, SD for the trained groups (Fig. 7*B*). Each of these group-unique means of the F-revF peak separation was significantly greater (*p* = 0.0283 for the naïve group and *p* = 0.00071 for the trained group; Table 4) than the mean spontaneous separation of the two *maxP*s obtained by repeating the F twice (9.66 ± 9.42, SD, over seven hemispheres of three trained and four naïve animals). However, these peak-to-peak distances were not significantly different between the animal groups (Fig. 7*B*, *p* = 0.386; Table 4).

**Table 4.** Comparison between separation distance of *maxP*s evoked by sounds and internal fluctuation distance

	Euclidian distance betweenF- and revF-evoked *maxP*s of activation		Internal fluctuation, distance of *maxP*s evoked by repeating F		Comparison of separation distance of stimulus-evoked *maxP*s to internal fluctuation distance		Comparison of separation distance of F-evoked and revF-evoked *maxP*s between animal groups	
Animal group	Coordinate-based distance	Number ofhemisphere s used	Coordinate-based distance	Number ofhemispheres used	Type of test	p values	Type of test	*p* value
Trained	33.6 ± 15.8	12	9.66 ± 9.42	7	Welch test	7.09E-04	Welch test	0.3858
Naïve	26.8 ± 21.3	12	Welch test	0.0283

### Comparison of activation between animal groups

Stimulation with the F segments consistently activated the auditory cortex more strongly than stimulation with the revF segments, with the evoked *maxP*s (Fig. 7*A*) being significantly different for both the trained (0.212 ± 0.063, SD vs 0.116 ± 0.029, SD; *p* = 8.74E-05; Table 5) and the naïve groups (0.199 ± 0.0600, SD vs 114 ± 0.026, SD; *p* = 1.97E-04; Table 5). The comparison of these *maxP*s between the animal groups showed that the F-evoked AI *maxP* was slightly larger for the trained group than for the naïve group, but this difference did not reach a significance level (Fig. 7*A*, *p* = 0.621; Table 6). Similarly, the revF-evoked AI *maxP*s were not statistically different between the animal groups (Fig. 7*A*, *p* = 0.872; Table 6).

**Table 5.** Comparison between magnitudes of *maxP*s evoked by F stimulation and revF stimulation, dF/Fmax %

Animal group	Number ofhemispheres used	MaxPs of activation evoked byF and revF stimulation		Comparison between sound types	
		F	revF	Type of test	*p* values
Trained	12	0.212 ± 0.063	0.116 ± 0.029	*t* test	8.74E-05
Naïve	12	0.199 ± 0.060	0.114 ±0.026	*t* test	1.97E-04

**Table 6.** Comparison between *maxP*s of activation for different animal groups, dF/Fmax %

Sound type	Animal group				Comparison between animal groups	
	Trained	Number ofhemispheres used	Naïve	Number ofhemispheres used	Type of test	*p* values
F	0.212 ± 0.063	12	0.199 ± 0.060	12	Welch test	0.621
revF	0.116 ± 0.029	12	0.114 ± 0.026	12	Welch test	0.872

### Comparison of the activation contrasts between animal groups

Magnitude of response signals varied substantially among hemispheres; therefore, we normalized the response signals evoked by the revF to those evoked by the F for each hemisphere. Means of these hemisphere-unique contrasts were compared between animal groups. The ratios of the revF-evoked *maxP* relative to F-evoked *maxP* within the AI (RmP/FmP) were not significantly different between the naïve and trained animal groups (0.600 ± 0.140, SD vs 0.574 ± 0.159, SD, respectively; *p* = 0.675; Fig. 7*C*, left; Table 7). However, if the cortical location where stimulus sounds activated the AI maximally (corresponding to the F-evoked *maxP*) was specifically concerned, the ratio of the revF-evoked *tempM* at this location relative to the F-evoked *maxP* (RtM at FmP/FmP) was significantly smaller in the trained than in the naïve groups (0.348 ± 0.116, SD vs 0.449 ± 0.088, SD, respectively; *p* = 0.0264; Fig. 7*C*, right; Table 7), although these revF-evoked *tempMs* at the F-evoked *maxP* location themselves were not significantly different between the animal groups (0.0711 ± 0.023, SD vs 0.0873 ± 0.027, SD, *p* = 0.129; Fig. 7*A*; Table 8). Thus, the reduced contrast for the trained animals indicates that the neuronal population most sensitive to a given natural sound was more strongly suppressed by its time-reversed unnatural counterpart after conditioning to the natural version.

**Table 7.** Comparison between contrasts of revF-activation relative to F-evoked activation for different animal groups

Type of activation contrast	Number of hemispheres used	Ratio of revF-evoked maxP relative to F-evoked maxP		Comparison between animal groups	
		Animal group		Type of test	*p* values
		Naïve	Trained		
RevF-evoked maxP/F-evoked maxP	12	0.600 ± 0.140	0.574 ± 0.159	Welch test	0.6745
TempM of revF-evoked activation at F-evoked maxP/F-evoked maxP	12	0.449 ± 0.088	0.348 ± 0.116	Welch test	0.0264

### Activation of the VA in the trained and naïve groups

The F-evoked activation peaks first emerged in the dorsal region soon after the sound onset and shifted ventrally to the central AI. The peaked activation then propagated further ventrally/anteroventrally to the VA with minimum attenuation in amplitude for the trained group. Occasionally, the activation propagated further ventrally beyond the ventral edge of the image frame. When the activation was mapped at the threshold larger than the 6 SD of the means of spontaneous activities, the peaked activation was tended to spread to the VA more frequently for the trained (8 out of 10 hemispheres; Fig. 8, left) than for the naïve group (4 out of 8 hemispheres; Fig. 8, right), though they were not statistically significant (two-tailed, *p* = 0.321; Table 9). The F-evoked VA *maxP*s themselves were not statistically different between the naïve and trained groups (0.169 ± 0.074, SD vs 0.208 ± 0.088, SD; *p* = 0.1429; Table 10). However, the normalized *maxP*s relative to those in AI were significantly different between animal groups; the ratios of the F-evoked VA *maxP* relative to the F-evoked AI *maxP* were 0.665 ± 0.174 (mean ± SD) for the naïve group versus 0.912 ± 0.177 (mean ± SD) for the trained group (*p* = 0.0097; Fig. 7*D*; Table 10).

In contrast, at the threshold used for the F activation maps, the revF stimulation did not evoke the peaked activation within the VA (*n* = 18) for either animal group (Fig. 9, data not statistically analyzed).

**Table 8.** Comparison of tempMs of revF-evoked activation recorded at the location of F-evoked maxP between different animal groups, dF/Fmax %

Animal group	Number of hemispheres used	RevF-evoked tempM at F-evoked maxP	Comparison between animal groups	
			Type of test	*p* value
Trained	12	0.0711 ± 0.023	Welch test	0.129
Naïve	12	0.0873 ± 0.027		

**Table 9.** Comparison of occurrence of VA activation peaks between animal groups

Animal group	Number of hemispheres used	Number of hemispheres with VA peaked activation	Number of hemispheres without VA peaked activation	Comparison between animal groups	
				Type of test	*p* value
Naïve	8	4	4	Fisher’s exact probability test, two-tailed	0.3213
Trained	10	8	2		

**Table 10.** Comparisons of F-evoked *maxP*s in the VA and AI between trained and naïve animal groups

Animal group	Number of hemispheres used	MaxP in VA	Comparison of VA peaks between animal groups		MaxP in AI	MaxP in VA/maxP in AI (normalized)	Comparison of VA-AI peak ratios between animal groups	
			Type of test	*p* value			Type of test	*p* value
Trained	10	0.208 ± 0.088	Welch	0.1429	0.224 ± 0.061	0.912 ± 0.177	Welch	0.0097
Naïve	8	0.169 ± 0.074			0.228 ± 0.053	0.665 ± 0.174		

## Discussion

### Cortical activation evoked by temporally asymmetric sound pairs

The trial-unique *maxP*s of AI activation were significantly smaller for revF than for F stimulation (Fig. 7), indicating that the temporal asymmetry of envelopes affects the synaptic excitation. In addition to the magnitude difference, the latency to the *maxP* was prolonged with a greater intertrial fluctuation for revF than for F stimulation (Fig. 6). This response asymmetry may be due to the envelope transition effects on onset responses (Heil, 1997a,b). The response magnitude and latency are functions of the first derivative of the rise-time transition to peak pressure. Thus, the discharge rate is higher when the instantaneous onset slope of envelope is steeper and the first-spike latency is shortened when the acceleration of peak pressure is increased. Ramped-down sounds generated by applying convulsive force against hard materials, like the F of the present study, are characterized by a quick rise and slow decay of the envelope, while ramped-up sounds, such as the revF, are characterized by the opposite envelope configuration. Therefore, it is likely that the revF evokes lower spike discharge rates and longer peak latencies than the F.

The stimulus asymmetry was also reflected by the location of the *maxP*s in the AI. The discrete *maxP*s of activation evoked by F and revF stimulation were separated more widely than the two *maxP*s evoked by repeating the F twice were segregated (Fig. 7*B*). This significant separation of the two *maxP*s indicates that a neuronal population activated maximally by the normal forward sound is not the population that is activated maximally by its reversed counterpart despite their identical spectrum content.

The propagation of activation from the dorsal to ventral regions of the AI, as revealed by F stimulation, was not fully reproduced by revF stimulation. The so-called isofrequency axis is oriented dorsoventrally, roughly perpendicular to the anterocaudal frequency gradient in the AI of the guinea pig (Hellweg et al., 1977; Redies et al., 1989). It is known that AI pyramidal neurons located along the isofrequency axis are interconnected through their horizontal collaterals (Ojima et al., 1991). Thus, the absence of the dorsoventral propagation of activation by the revF stimulation may be a reflection of weak or no interactions between AI cortical neurons through their intrinsic horizontal connections. An alternative interpretation is also possible. Our results may reflect disturbance of gradually delaying thalamocortical transmission along the AI isofrequency axis (Taniguchi et al., 1992; Wallace et al., 2000; Nishimura et al., 2007). Regardless, either hypothesis would lead to a deteriorated temporal sequence of activation peaks across the AI.

### Modification after conditioning to wideband natural sounds

Learning did not significantly affect the magnitude of activation peaks within the AI (Fig. 7*A*). Naïve animals had never been exposed to either the F or revF before testing, whereas trained animals had been intensively exposed to the F. Despite this difference in experience, the *maxP*s of F- and revF-evoked activation were almost the same whether animals were trained or not. This finding appears to contradict the well-known frequency-specific plasticity, but suggests that the learning of wideband sounds, such as the present F, may not augment the responsiveness to particular frequencies, probably because multiple frequency components would be used as spectral cues during conditioning. Instead, learning increased the contrast of synaptic responses evoked at the AI location that was activated maximally by the temporally asymmetric sound pair. It implies that when the neuronal population that is maximally activated by a forward sound is more vigorously suppressed by its asymmetric counterpart after learning (Fig. 7*C*). Conditioning to a particular temporal structure modifies network to be tuned preferentially to that envelope, and this modified network is now more resistant to be activated by the asymmetrically degraded temporal structure.

### Activation spread into the VA

Time course of the activation maps showed that the F-evoked activation extended from the dorsal to central region of the AI and then propagated to the VA (Figs. 3*B*, 4*A*). This propagation of F-evoked activation to the VA occurred more robustly after training, resulting in the greater VA-to-AI peak activation ratios for the trained animal group (Fig. 7*D*). Note that since the naïve animals had never heard the stimulus sound before testing, this VA activation might possibly be facilitated simply by the repeated exposure during training but not by learning. We cannot explicitly differentiate these possibilities. However, the finding that VA activation could be sporadically observed even in the naïve animals (Fig. 8) and also by pure tones stimulation in our routine mapping experiments (Horikawa et al., 2001) suggests that neither of the above possibilities may be a critical requisite for induction of the robust VA activation. We rather claim involvement of the emotive valence of training itself. During the competitive training, animals competed each other for food and frequently blocked the competitor to access food (Ojima and Horikawa, 2016), thus being in a strongly aggressive state. Aggression is known to be a highly emotional state (Albert et al., 1989; Nelson and Trainor, 2007). Therefore, it is expected that the neural network of emotion together with the reward system would be activated during the conditioning. Emotional learning is mediated through the corticoamygdala pathway (LeDoux, 1992; Ono et al., 1995; Phelps and LeDoux, 2005). Anatomically, the primary sensory cortex generally does not project to the amygdala, while the higher association cortices, via primary-to-association corticocortical connections, project to the amygdala (Grosso et al., 2015). For rats, networks for this processing include the following auditory connections (Romanski and LeDoux, 1993); the TE1 (corresponding to the AI) projects to the area just ventral to it (TE1v) and to other fields further surrounding the TE1v (i.e., TE2c and TE3v), and these nonprimary fields give rise to the corticoamygdala projection. Although no comparative data have been available, the VA of the guinea pig is possibly homologous to the TE1v on the basis of its topographical and connectional relationships with the AI (Wallace et al., 2002). The comparisons of activation strength between the trained and naïve animals revealed the stronger F-evoked VA activation after the competitive training (Fig. 7*D*). Altogether, the enhanced emotional state, the possible homology of the VA to rat not-AIs and their connections to the amygdala suggest intriguing implications for the role of emotional state and amygdala in perceptional leaning.

### Comparison to the previous studies

On the basis of spike counts, comparisons of neuronal activation evoked by natural calls and their time-reversed versions have been made for bird songs (McCasland and Konishi, 1981; Margoliash, 1983), marmoset twitter calls (Wang et al., 1995; Wang and Kadia, 2001) and cat meow calls (Gehr et al., 2000). Comparisons were also made between the artificial sinusoids of temporally asymmetric envelopes (Lu et al., 2001a,b; Wang et al., 2014). Interestingly, the conclusions from the studies using the natural and artificial sound types were not consistent. For animal songs/calls, neurons were generally more responsive to the natural versions than their time-reversed counterparts whether animals were anaesthetized (Wang et al., 1995; Gehr et al., 2000) or awake (McCasland and Konishi, 1981; Margoliash, 1983). Thus, the differences in the response magnitude may reflect differences in innate network or past experience. For the artificial sounds, a larger proportion of neurons preferred ramped-up sinusoids to ramped-down sinusoids (Lu et al., 2001b; Wang et al., 2014). This response preference was apparently opposite to our present result, which demonstrated a stronger activation with the ramped-down F than with its time-reversed revF. However, when we closely inspected the relevant responses evoked by the stimuli that were comparable in duration and envelope shape to ours and presented in the similar manner (see Figure 4 in Wang et al., 2014), the ramped-down sinusoids appear to generate larger responses than the counterparts if the discharge rates are measured only during the sound-on period comparable to ours (i.e., 80 ms).

Voltage-sensitive dye signals more likely reflect local field potentials (LFPs) than spiking of single neurons. Fractional changes in light intensity (dF/Fmax) are in proportional to changes in the membrane potential averaged across all neural elements under individual sensors (Chemla and Chavane, 2010). Their signal-to-noise ratio, however, is generally worse than that of the LFPs (London et al., 1989; Prechtl et al., 1997), since optical signals depend on many biological and technical factors. The γ-band oscillations in LFPs are stimulus dependent and engaged in coding sensory objects (Herrmann et al., 2010). In the auditory domain, presentations of single clicks, pure tones, and amplitude-modulated sounds generally evoke γ-band oscillations in a phase-locked manner (Galambos et al., 1981; Pantev et al., 1991; Franowicz and Barth, 1995). In addition, natural sounds including human voices (Crone et al., 2006) and animal calls (Medvedev and Kanwal, 2004) have been reported to evoke γ-band oscillations depending on the meaningfulness of those sounds. Thus, when communication calls were temporally reversed, the evoked γ-band oscillations were generally attenuated, as shown for the bat communication calls (Medvedev and Kanwal, 2008). Like LFPs, the optical signals of voltage-sensitive dyes report the population activity of neural elements, but only low frequency oscillations of optical signals (around 10 Hz) have so far been observed in a limited number of studies *in vivo*, for example, from the turtle olfactory bulb (Lam et al., 2000) and also from the rat visual and turtle somatosensory cortices (London et al., 1989; Prechtl et al., 1997). In contrast to these recordings, our sound-evoked optical signals, band-passed at a frequency range of 4.0-40 Hz (overlapped partially with the typical 30- to 80-Hz γ-band range) did not exhibit prominent oscillating components that had been previously found within the LFPs of the AI. Preliminary measurements confirmed this by filtering the unfiltered original signal at the above-mentioned typical γ-band range and revealed a few cycles of fluctuation that were very small in amplitude and strictly coincident with the slow-wave large response. For now, it is unclear why γ-band components could not be observed in our optical signals. Further examination will be needed to interpret this apparent absence of auditory oscillatory components in the optical signals.

We assume that the activation patterns of voltage-sensitive dye signals reflect the network’s processing of instantaneous spectral content within sounds, probably because of the relatively slow response times. For the network to perform such rapid processing, different populations of neurons must be continuously recruited because of their refractory periods. This rapid recruitment of different neuronal populations may be reflected in the propagation of activation along the AI iso-frequency axis that likely represent temporally varying spectra within complex sounds (Langner et al., 2009; Brewer and Barton, 2016).

## Synthesis

Reviewing Editor: Darcy Kelley, Columbia University Decisions are customarily a result of the Reviewing Editor and the peer reviewers coming together and discussing their recommendations until a consensus is reached. When revisions are invited, a fact-based synthesis statement explaining their decision and outlining what is needed to prepare a revision will be listed below. The following reviewer(s) agreed to reveal their identity: Jagmeet Kanwal

General comments

There are very few optical imaging studies of the auditory cortex where neural activity of hundreds of thousands of neurons can be directly and simultaneously mapped on a large scale. This makes the study novel and the results important for understanding how different cortical regions contribute to the representation and encoding of complex sounds. There are two aspects to the findings. One is that there is an asymmetry to the spatiotemporal activation pattern for “:ramped” vs. damped sounds (with temporally asymmetric amplitude envelops). The second has to do with the spread of the activation pattern to the VA field in the belt region for sounds to which the animal was reward-trained. In this manuscript, the authors use voltage-sensitive dye imaging to determine the areas of auditory cortex that are activated by “Forward” damped sounds and their reversed versions in naive and trained animals. They show that cortex is more strongly activated by forward sounds. Training suppresses the response to reversed sounds at the location of the maximum peak for forward sounds. The data show a large asymmetry in the cortical representation of forward vs. reversed sounds using a technique that can reveal the bias of large neuronal populations, and will be of interest to auditory neuroscientists. The training paradigm is novel and seems effective, although this seems to have been previously published.

Interpretation of the data can be based upon single unit recordings or on multiunit cortical activity for ensembles of neurons in the form of evoked potentials. Averaged neural activity visualized with voltage-sensitive dyes sampled at 2 ms intervals more likely reflects local field potential activity. In this context, there is no mention of the literature on gamma oscillations that are known to be triggered by the presentation of meaningful (communication) sounds both in animals (e.g., bats) and in humans. Presentation of the same sound in reverse is known to decrease the magnitude of the evoked gamma oscillation signal in the high-frequency range. These findings along with single unit data need to be carefully considered when interpreting the results of the study and may contribute especially to subthreshold activation. The authors suggest that single unit data on temporally varying sounds explains the spread of activity over the cortical surface and is a result of rapidly evolving spectral content of the sound. While this may be true to some extent, the perception of the “conditioned” meaning of the sound may activate a different set of late-responding cortical neurons via cortico-subcortical loops.

At a more basic level, one needs to consider what exactly is happening when a sound is paired with a reward. This sound is taking on meaning for the animal, in this case, being tagged with positive valence or an emotive value. Many communications sounds in animals similarly have emotive relevance that may be learnt during development. Therefore, one interpretation of the data is that ventral regions of the cortex encode or represent sounds which have emotive value, regardless of whether they are communication or environmental sounds. This may depend on the connections of this region of the cortex with subcortical structures such as the amygdala and/or the nucleus accumbens; including this in the discussion will make the findings more relevant and appeal to a wider audience. In fact, this is likely the more important and novel result of this study. As stated by the authors on line 61, effect of time-reversing sounds has been demonstrated in several animal species. What was not said was that much or all of these data in different species come from single unit studies.

The presentation and writing needs substantial improvement. The abstract and introduction needs to be rewritten. A clear hypothesis is not proposed and the major findings are not adequately stressed. Some figures need more clear explanation, and statistical analyses are appropriate. Some of the data shown in statistical tables, however, can be incorporated within the text.

Specific comments

1) Arbitrary setting of threshold is problematic. The authors note that they determine the amplitude of the maximum peak, and for Forward sounds, set the threshold to 50% of this value. However, for reversed sounds, the threshold was reduced to a trial-unique, arbitrary value. This is problematic for two reasons:

a) It is difficult to evaluate whether the peak detected is “real” or is just background noise. Judging from the traces in Figure 8, there seems to be a fair amount of variability in the background signal. To avoid spurious peak detection, it would be better to define the threshold as a multiple of the standard deviation of the background noise (3*SD or 5*SD, for example) and keep the definition of threshold consistent.

b) The current method of arbitrarily lowering the threshold until a peak is detected might be the reason for the observed longer and more variable latency for revF sounds - It might create spurious variability in the data.

2) Error bars/significance?

The use of an arbitrary threshold to measure “subthreshold” responses raises the question of whether these responses are statistically significant. How do the authors evaluate if they observed a significant response, whether sub- or supra-threshold?

3) Description of analysis needs to be clearer.

On Page 8/Line 170, it is stated that a stimulus unit composed of a sound-on period of the 4-segment train was played 4 times. Does this mean that the averaging is over 4 sounds, or 16 sounds? However, later, in Page 10/Line 235, the authors state that analyses were focused on response signals evoked by only the first segment. So is the final analysis based on responses to 1 or 4 sounds?

4) In the Abstract, a central claim is: Line 13 “... if the location where the F-evoked maximum peak was recorded was stimulated by the revF, the revF-evoked activation was significantly smaller in the trained group than in the naive group”. But in Fig 5A, Rtm at Fmp in Naive animals and Rtm at Fmp in Trained animals is marked as not significant.This claim in the abstract comes from 5B after normalizing, but this does raise the question of the robustness of the claim as stated. Coupled with point (2) above, it is critical to know if the authors are comparing meaningful signals here. Line 14: “... Location where the F-evoked maximum peak was recorded was stimulated by the rev F ...” not clear how a cortical location can be directly stimulated by sound. A sound can be presented or the cortical location can be directly electrically stimulated. Also In the first sentence of the abstract, it should be made clear that authors are referring to a complex sound; simple sounds (pure tones) are heard the same when played back in reverse.

5) There seems to be high variability in the time course of activation (not latency). For example, in Fig. 2A, the entire response takes about 40ms for F sounds. But in Fig 2B, the revF response duration seems to be closer to 80 ms, and in 2C it is close to 60 ms. What is the cause of this variability?

6) Labeling of some axes/color scales are non-standard and confusing.

For example, in Figure 2:

a) There are colorbars, but no markings to determine what amplitude the colors represent

b) The pt = (x,y) does not add any information

c) Positive deflection in trace has negative peak value

d) In Panel A, subpanel b, why is the top trace in red color?

7) The authors do not note if they encountered photobleaching and if long-term trends were regressed out prior to analysis. How long did it take to image each hemisphere ?

8) Why are the responses to tones >2KHz much weaker than responses to <2KHz in Figure 7 ? Is the variability seen here indicative of the limitations of the method? A scale bar here will be useful.

9) Line 6: change to “spectrum content.”

10) The use of the term “ramped” does not appropriately contrast with “damped”, since a parameter can either be ramped up or down. It is better to either say “ramped-up” or “amped” to appropriately contrast with “damped”.

11) How do variations in the levels of anesthetics used affect cortical activity? Was this tested in a control or is established elsewhere?

12) Sentence on line 361 appears to contradict the next section (5) of the results. Please elaborate/clarify. May be good to state in 3 to 4 sentences at the beginning of results section how the results are organized and what the main results are.

13) May be useful to move figure 7 showing cortical activation to tones towards the beginning and include a schematic identifying the brain regions (mostly auditory cortex) in relation to the major blood vessels as visualized in the optical recordings.

14) The separation of left versus right hemispheres in figure 6 is not clear.
